# Multiple R1 margins are associated with shorter overall survival: national register-based study on pancreatic cancer

**DOI:** 10.1093/bjsopen/zrag155

**Published:** 2026-10-16

**Authors:** Patrik Larsson, Carlos Fernández Moro, Melroy D’Souza, Bobby Tingstedt, Ernesto Sparrelid, Poya Ghorbani

**Affiliations:** Division of Surgery and Oncology, Department of Clinical Science, Intervention and Technology, Karolinska Institutet, Karolinska University Hospital, Stockholm, Sweden; Department of Laboratory Medicine, Division of Pathology, Karolinska Institutet, Karolinska University Hospital, Stockholm, Sweden; Department of Clinical Pathology and Cancer Diagnostics, Karolinska University Hospital, Stockholm, Sweden; Division of Surgery and Oncology, Department of Clinical Science, Intervention and Technology, Karolinska Institutet, Karolinska University Hospital, Stockholm, Sweden; Department of Surgery, Clinical Sciences Lund, Skåne University Hospital, Lund University, Lund, Sweden; Division of Surgery and Oncology, Department of Clinical Science, Intervention and Technology, Karolinska Institutet, Karolinska University Hospital, Stockholm, Sweden; Division of Surgery and Oncology, Department of Clinical Science, Intervention and Technology, Karolinska Institutet, Karolinska University Hospital, Stockholm, Sweden

**Keywords:** R status, margins of excision, overall survival, pancreatic neoplasms, pancreatic surgery, pancreatoduodenectomy, prognosis

## Abstract

**Background:**

Resection status is associated with overall survival among patients operated on for pancreatic ductal adenocarcinoma (PDAC). This study investigated whether the number of R1 margins is associated with shorter survival.

**Methods:**

Adult patients who underwent pancreatoduodenectomy for primary resectable PDAC between 2017 and 2024 recorded in the Swedish National Pancreatic Registry were included. Overall survival was analysed according to overall R status and the number of R1 margins. Overall survival was analysed using Kaplan–Meier estimates, Cox regression analysis for estimated adjusted survival time, and Laplace regression for analysis of percentile distribution.

**Results:**

Overall, 1043 patients were included, 398 patients (38.2%) with R0 and 645 patients (61.8%) with R1 margins. The adjusted median survival was 8.2 (95% confidence interval (c.i.) −12.0 to −4.3) months shorter for patients with R1 *versus* R0 margins, with a progressively shorter survival with a higher number of R1 margins. Specifically, compared with R0 margins, the adjusted median survival for one, two and three or more R1 margins was 4.6 (95% c.i. −10.2 to 1.0), 6.7 (95% c.i. −11.9 to −1.5), and 12.4 (95% c.i. −16.5 to −8.3) months shorter, respectively.

**Conclusions:**

Patients with R1 margins had an adjusted median survival that was 8 months shorter than patients with R0 margins. An increased number of R1 margins was associated with shorter survival. These results suggest that the current practice of reporting binary R status is insufficient, and future guidelines should consider revising this routine and adopt a standardized, detailed margin assessment protocol.

## Teaser

This study evaluated the association between the number of R1 margins and survival among patients undergoing surgery for pancreatic cancer. The study found a progressively shorter survival with an increasing number of R1 margins. The adjusted median survival was 4.6, 6.7, and 12.4 months shorter for one, two, and three or more R1 margins, respectively, compared with R0 margins.

## Introduction

Pancreatic cancer is one of the leading causes of cancer mortality^1–3^. The cornerstone for treating localized pancreatic cancer is surgery combined with chemotherapy^4^. Analysing the resected tumour provides the possibility of assessing several cancer characteristics, such as tumour node metastasis (TNM) and resection (R) status, as well as molecular profiling^5–9^. A short distance between tumour cells and the surface of the specimen (R1 resection status), has been shown to be associated with shorter overall survival compared with R0 resections^10,11^. Despite attempts to harmonize the definition of the R status, there are still differences between national routines in how R status is assessed^12,13^. Moreover, although most guidelines recommend measuring R status in several margins, current literature reports R status as a single binary composite variable (R0 or R1). It is unclear whether the R status holds more relevant information.

The aim of this study was to investigate how the R status is associated with the survival of patients who undergo pancreatoduodenectomy for pancreatic ductal adenocarcinoma (PDAC). The study tested two hypotheses: first, R1 status is associated with a shorter survival and a higher risk of recurrence than R0 (validation of the current practice to analyse R status); and second, there is an association between an increased number of R1 margins and shorter survival and a higher risk of recurrence.

## Methods

This cohort study was performed in accordance with the STROBE guidelines^14^ and was approved by the Swedish Ethical Review Authority.

### Study population

All data were collected from the Swedish National Pancreatic and Periampullary Cancer Registry. The registry was initiated in 2009, covering all centres performing pancreatic surgery in Sweden^15^. All pathological examinations follow the same national guideline^16^. The variables in the pathological report, including R status, were last updated in 2017. Patients were eligible for inclusion in the study if they were adult (age 18 years or older), had undergone pancreatoduodenectomy between 2017 and 2024, had a preoperative diagnosis of primary resectable pancreatic cancer (as per the definitions of the National Comprehensive Cancer Network^17^), and had a postoperative pathological diagnosis of PDAC. Exclusion criteria comprised a preoperative diagnosis of metastatic disease and neoadjuvant chemotherapy. Neoadjuvant chemotherapy was considered an exclusion criterion for the following reasons. First, patients treated with neoadjuvant chemotherapy who undergo surgery have had an additional treatment compared with patients not treated with neoadjuvant chemotherapy. These patients treated with neoadjuvant chemotherapy have had an additional selection that could affect the outcome with a risk of confounding. Second, neoadjuvant treatment may have influenced the exposure (R status) and may be a risk for additional confounding. Third, at the beginning of the study period, there was a discussion on how to assess R status among patients with neoadjuvant chemotherapy that suggested a higher risk of missing R status values at the start of the study period among patients who have received neoadjuvant chemotherapy. Thus, neoadjuvant chemotherapy was considered as an exclusion criterion to minimize the risk of incorporating additional confounding.

### Exposures and measures

The study questions were defined using the PICO (Population, Intervention, Comparison, Outcome) framework^18^. The study sought to answer two questions: the effects of overall R status (PICOS-1) and the number of R1 margins (PICOS-2) on overall survival compared with a negative overall R status (R0). In both cases, the population comprised adult patients with a primary resectable pancreatic cancer who underwent pancreatoduodenectomy with a pathology report of PDAC; the primary outcome was overall survival from time of surgery and secondary outcomes were recurrence and major postoperative complications (defined as Clavien–Dindo grade ≥ IIIa).

### Variables and definitions

The definitions for R status followed the Swedish national pathology guideline^16^. The Swedish National Pancreatic and Periampullary Cancer Registry holds data on R status, both overall R status as well as data on the R status for eight different margins (posterior resection surface, superior mesenteric vein (SMV) groove surface, superior mesenteric artery (SMA) surface, pancreatic transection margin, bile duct margin, duodenal/gastric resection margin, anterior surface, and, if a venous resection was performed, involvement of the vein resection margin). For all resection margins, except the anterior margin, R1 is defined as tumour cells < 1 mm from the transection/dissection surface and R0 as ≥ 1 mm. For the anterior resection surface, the distance for R1 is 0 mm and for R0 > 0 mm. The overall R status is defined as R1 if any of the measured margins are categorized as R1 and as R0 if all margins are R0. The definitions of R1 and R0 in the present study followed these national criteria.

The primary outcome in this study was overall survival, measured from the time of surgery. Secondary outcomes were recurrence, defined as a registered recurrence in the Swedish National Pancreatic and Periampullary Cancer Registry, and postoperative complications, defined as Clavien–Dindo grade ≥ IIIa^19^. Recurrence is registered in the national registry as a dichotomous measure (yes/no). The registry does not hold information on the location of the recurrence. The national routine for postoperative follow-up for detection of recurrence is computed tomography every 6 months for the first 2 years and then individualized follow-up thereafter^16^. Other variables included in the registry data are patient age, sex, body mass index (BMI), preoperative Eastern Cooperative Oncology Group (ECOG) performance status^20^, preoperative American Society of Anesthesiologists (ASA) physical status^21^, preoperative bilirubin, preoperative serum carbohydrate antigen 19-9 (CA19-9), peroperative bleeding, the centre performing the surgery, venous resection, arterial resection, tumour size, total number and number of positive regional lymph nodes, and total number and number of non-regional lymph nodes.

The follow-up period was defined as the time from the date of surgery until the date of death, loss to follow-up, or the end of study (30 June 2025), whichever came first. The interval for postoperative follow-up is not registered in the Swedish National Pancreatic and Periampullary Cancer Registry.

#### Missing data

Variables with missing data were explored. The missingness of data was evaluated. In the event that the R status of a margin was categorized as missing but the overall R status was categorized as R0, the missing margin was categorized as R0. This was based on the prerequisite that the overall R status can only be R0 if all margins have been categorized as R0^16^. Patients with no data on R status (five patients in total) were excluded from the study. There was a large proportion of missing data on recurrence and date of recurrence. This hindered any statistical analysis of recurrence. Evaluation of the remaining variables indicated data were missing at random and were considered as such for analysis. Patients lost to follow-up were censored at the last date of known outcome.

### Statistical analysis

Statistical analyses were performed using Stata^®^ release 17 (StataCorp, College Station, TX, USA).

Categorical variables are presented as counts with percentages, whereas continuous variables are presented as the median with interquartile range (i.q.r.). R status was categorized as R0 and R1 for PICOS-1. For PICOS-2, which evaluated the effects on survival of the number of positive margins, R status was categorized as R0 and one, two, and three or more R1 margins. The statistical models included confounders that were decided *a priori* and visualized using a directed acyclic graph (*Fig. S1*)^22^. The covariates included in all regression models (that is, Laplace, logistic and Cox regression) were patient age, sex, BMI, ASA grade, ECOG performance status, the centre performing the surgery, and tumour size as listed in the pathology report.

Analyses for PICOS-1 and PICOS-2 were performed as complete case analyses (main analysis). A sensitivity analysis was performed using multiple imputation for exposure (R status) and confounders under a missing-at-random assumption. Multiple imputation was performed using chained imputation^23^. The imputation model included the exposure (resection margin), all confounders (sex, age at surgery, region performing surgery, BMI, ASA grade, ECOG performance status, tumour size), auxiliary variables (positive lymph nodes, previous endoscopic retrograde cholangiography, preoperative bilirubin, preoperative CA19-9, peroperative bleeding), the outcome (survival status), and log-transformed follow-up time. The imputation was generated with 20 imputed data sets and combined effect estimates using Rubin’s rules^24,25^. Multiple imputation was used because it gives estimates of the missing values based on the known data^26^. Because the multiple imputaion is based on the missing-at-random assumption, the missingness of the data was evaluated which suggested that the data were missing at random. Outcomes were compared using χ^2^ tests. Postoperative complications were investigated with logistic regression analysis.

Median follow-up was calculated using the reverse Kaplan–Meier method. Unadjusted survival distribution was analysed with the Kaplan–Meier method using log-rank tests to determine the significance of differences. Adjusted survival distribution was analysed with two different approaches. First, Cox proportional hazard regression analysis was used to analyse the association between R status and overall survival, adjusted for the aforementioned confounders. The results are presented in an estimated survival graph and as hazard ratios (HRs) with their 95% confidence interval (c.i.). The proportional hazards assumption was assessed graphically using the Schoenfeld residual test and the Grambsch–Therneau test. Second, Laplace regression was used to estimate adjusted differences in survival time according to R status and at the 25th, 50th (median), and 75th percentiles of the survival distribution. Standard errors and 95% c.i. values were obtained using 1000 bootstrap replications with fixed random seed^27^. Laplace regression was included because it can analyse differences in adjusted percentile survival. This allows comparison of the adjusted median survival time, which, for many clinicians, may be more clinically understandable and relevant than the HR.

## Results

### Baseline characteristics

In all, 1043 patients (398 (38.1%) with R0, 645 (61.8%) with R1) were included in the study. There were no major differences in sex, age, co-morbidities, and BMI between the R1 and R0 groups (*Table 1* and *Table S1*). Compared with the R0 group, more patients with R1 underwent venous resection (35 (8.8%) *versus* 207 (32.1%), respectively) and tumour size was larger in the R1 group (median 30 (i.q.r. 25–40) *versus* 35 mm (i.q.r. 28–43) mm, respectively). There were no major differences between the groups in spread to regional and non-regional lymph nodes (*Table S2*). There were only minor differences between centres in all included variables.

**Table 1:** Background characteristics of all patients and stratified according to overall resection margin status

	All patients (*n* = 1043)	R0 (*n* = 398)	R1 (*n* = 645)
Age (years), median (i.q.r.)	71 (65–77)	71 (64–76)	71 (65–77)
**Sex**			
Female	532 (51.0%)	196 (49.2%)	332 (51.5%)
Male	511 (49.0%)	202 (50.8%)	313 (48.5%)
**BMI (kg/m^2^), median (i.q.r.)**	25 (22–28)	25 (23–28)	25 (22–28)
Missing	26 (2.5%)	13 (3.3%)	13 (2.0%)
**ASA grade, median (i.q.r.)**	II (II–III)	II (II–III)	II (II–III)
Missing	4 (0.5%)	0	4 (0.6%)
**ECOG PS, median (i.q.r.)**	0 (0–1)	0 (0–1)	0 (0–1)
Missing	12 (1.2%)	0	12 (1.9%)
**CA19-9 (kU/l), median (i.q.r.)**	168 (48–557)	125 (34–405)	187 (57–657)
Missing	216 (20.7%)	105 (26.4%)	111 (17.2%)
**Peroperative bleeding (ml), median (i.q.r.)**	350 (200–600)	350 (200–600)	350 (200–600)
Missing	4 (0.4%)	0	4 (0.6%)
**Venous resection**	243 (23.3%)	35 (8.8%)	207 (32.1%)
Missing	75 (7.2%)	41 (10.3%)	33 (5.1%)
**Artery resection**	4 (0.4%)	1 (0.3%)	3 (0.4%)
Missing	158 (15.1%)	78 (19.6%)	77 (11.9%)
**Tumour size total (mm)**	34 (27–42)	30 (25–40)	35 (28–43)
Missing	81 (7.8%)	39 (9.8%)	41 (6.4%)

Values are *n* (%) unless otherwise stated. i.q.r., interquartile range; BMI, body mass index; ASA, American Society of Anesthesiologists; ECOG PS, Eastern Cooperative Oncology Group performance status; CA 19-9, serum carbohydrate antigen 19-9.

### Distribution of R1

There were 398 patients (38.1%) with R0 status, 252 patients (24%) with one R1 margin, 161 patients (15%) with two R1 margins , and 193 patients (18%) with three or more R1 margins (*Table S3*). Among all patients, 414 (40%) were R1 towards the SMV, 253 (24%) were R1 towards the SMA, and 215 (21%) were R1 on the posterior surface (*Table 2*). There was no association between R1 of a specific margin and shorter overall survival compared with other margins (data not shown).

**Table 2:** Number of patients with R1 per margin (*n* = 1043)

	No. of patients (%)
R1 posterior	215 (20.6%)
R1 SMV groove	414 (39.7%)
R1 SMA	253 (24.3%)
R1 anterior	74 (7.1%)
R1 pancreas transection margin	112 (10.7%)
R1 bile duct	43 (4.1%)
R1 duodenum/stomach	41 (3.9%)
R1 vein resection	49 (4.7%)

SMV, superior mesenteric vein; SMA, superior mesenteric artery.

### Outcome analysis, survival, recurrence, and complications

The median follow-up was 4.3 (i.q.r. 2.9–6.7) years, corresponding to a total of 2572 person-years of observation. Overall, 708 patients (68%) died during follow up (*Table 3* and *Table S4*). There was no difference in major postoperative complications for between patients with R1 to R0 (PICOS-1) or the number of margins with R1 (PICOS-2) (*Table 3*). The prevalence of Clavien–Dindo ≥ IIIa was 125 (19.4%) and 71 (17.8%) patients in the R1 and R0 groups, respectively. In the statistical models, in the adjusted models, there was no difference in major postoperative complications between patients with R1 and R0. The odds ratio when comparing patients with R1 to those with R0 was 1.15 (95% c.i. 0.81 to 1.63; *P* = 0.433). Cancer recurrence was registered for 328 patients (31%), although there was a large proportion of missing values for time to recurrence (missing for 990 patients, 94.9%). This hindered statistical modelling of time to recurrence. In all survival analyses, patients with R1 had shorter survival than patients with R0. The median survival was 34.3 (95% c.i. 29.0 to 39.4) months for patients with R0, compared with 25.0 (95% c.i. 22.4 to 28.0) months for patients with R1 (*Fig. S2*). Survival decreased progressively with an increasing number of R1 margins, being 29.5 (95% c.i. 23.8 to 32.1) months for patients with one R1 margin, 26.3 (95% c.i. 21.8 to 33.4) months for those with two R1 margins, and 20.2 (95% c.i. 16.5 to 25.0)  months for those with three or more R1 margins.

**Table 3:** Patient outcomes for all patients and stratified by overall resection margin status

Outcome	All patients (*n* = 1043)	R0 (*n* = 398)	R1 (*n* = 645)	P*
**Dead**	708 (67.9%)	245 (61.6%)	459 (71.2%)	0.001
**Recurrence**	328 (31.4%)	112 (28.1%)	215 (33.3%)	0.035
Missing	245 (23.5%)	89 (22.4%)	154 (23.9%)	
**Clavien–Dindo grade ≥** **IIIA**	197 (18.9%)	71 (17.8%)	125 (19.4%)	0.492
Missing	11 (1.1%)	2 (0.5%)	9 (1.4%)	

Values are *n* (%). *χ^2^ test.

Analyses of proportionality, both visual and with the Schoenfeld residual test, suggested proportional hazards. The adjusted predicted survival probability was shorter for patients with R1 than for those with R0 (*Fig. 1*; HR 1.51; 95% c.i. 1.27 to 1.79; *P* < 0.001). Analysis of the number of R1 margins (PICOS-2) revealed a progressively shorter survival with an increasing number of R1 margins, with an adjusted HR, relative to R0, of 1.29 (95% c.i. 1.05 to 1.60; *P* = 0.009) for one R1 margin, 1.44 (95% c.i. 1.15 to 1.82; *P* = 0.001) for two R1 margins, and 1.94 (95% c.i. 1.55 to 2.42; *P* < 0.001) for three or more R1 margins (*Table S5*). Sensitivity analysis with multiple imputation yielded similar results (*Table S6*).

**Figure 1:**
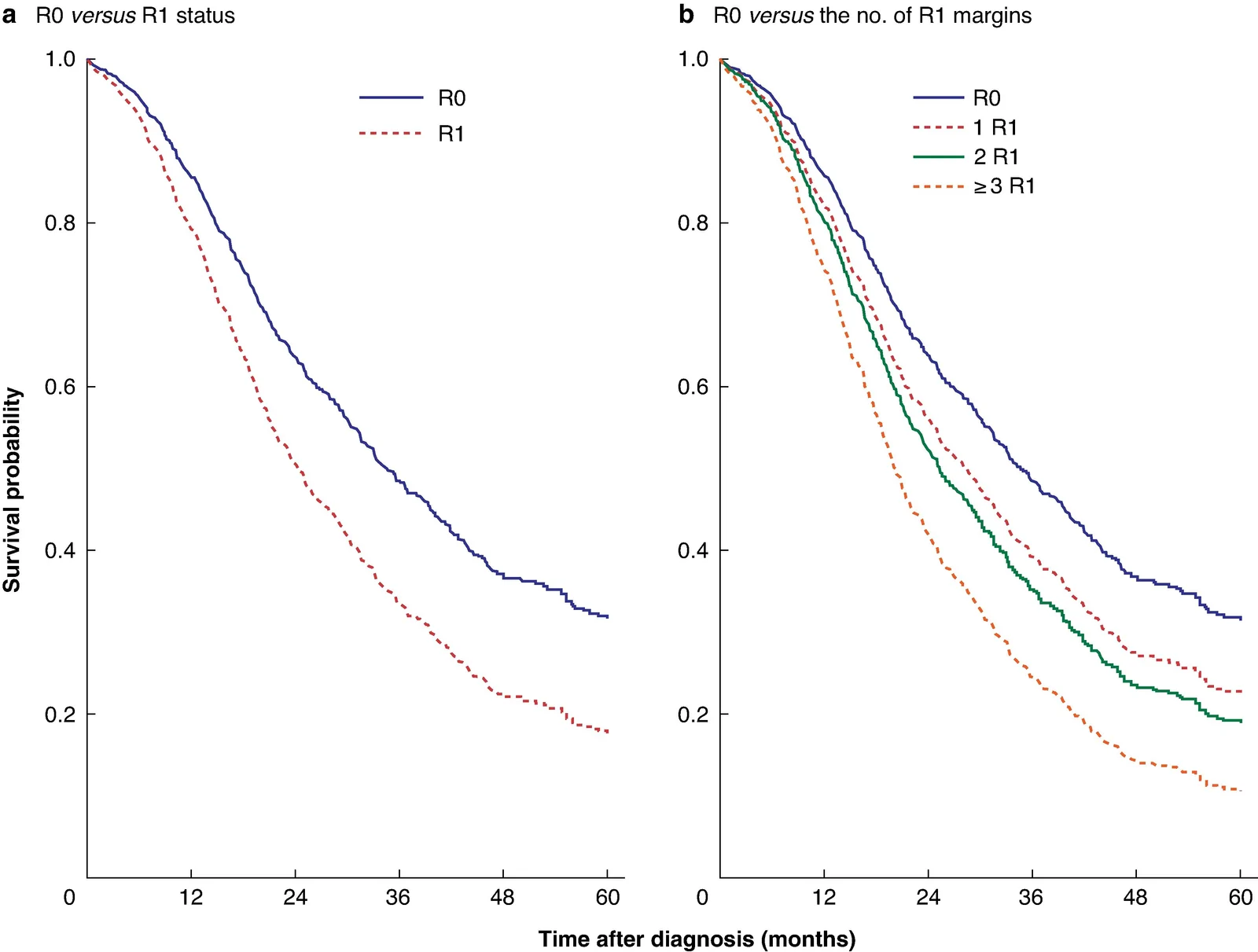
Estimated overall survival probability from adjusted Cox proportional hazard regression analysis from the time of surgery **a** Estimated overall survival probability for R0 (reference) *versus* R1 status. The hazard ratio (HR) for the R1 group was 1.51 (95% confidence interval (c.i.) 1.27 to 1.79; *P* < 0.001). **b** Estimated overall survival probability for R0 (reference) *versus* the number of R1 margins. HRs for one, two, and three or more R1 margins were 1.29 (95% c.i. 1.05 to 1.60; *P* = 0.019), 1.44 (95% c.i. 1.15 to 1.82; *P* = 0.002), and 1.94 (95% c.i. 1.55 to 2.42; *P* < 0.001), respectively. The following covariates were included in the adjusted model: age at surgery, sex, American Society of Anesthesiologists grade, Eastern Cooperative Oncology Group performance status, body mass index, tumour size, and centre performing the surgery.

Laplace regression analysis showed that the adjusted median survival was 8.2 months shorter for patients with R1 (95% c.i., −12.0 to −4.3) compared with patients with R0 (*Table 4*). There was progressively shorter survival with an increasing number of R1 margins (PICOS-2), with an adjusted median survival, relative to R0, 4.6 (95% c.i. −10.2 to 1.0) months shorter for one R1 margin, 6.7 (95% c.i. −11.9 to −1.5) months shorter for two R1 margins, and 12.4 (95% c.i.: −16.5 to −8.3) months shorter for three or more R1 margins. Similar patterns were observed at the 25th and 75th percentiles (*Table 4*).

**Table 4:** Laplace regression estimates for adjusted predicted survival time and differences at the 25th, 50th, and 75th percentiles

	Resection margin status	Adjusted predicted survival time* (months)	Adjusted difference† (months)
**PICOS-1**			
25th percentile	R0	17.9 (15.5, 20.3)	Reference
	R1	14.3 (12.9, 15.8)	−3.6 (−6.4, −0.9)
50th percentile	R0	34.2 (30.7, 37.7)	Reference
	R1	26.2 (23.9, 28.5)	−8.2 (−12.0, −4.3)
75th percentile	R0	62.1 (53.7, 70.6)	Reference
	R1	44.7 (40.7, 48.6)	−17.4 (−26.2, −8.6)
**PICOS-2**			
25th percentile	R0	17.7 (15.3, 20.2)	Reference
	1 R1	14.9 (12.8, 17.0)	−3.2 (−6.3, 0.0)
	2 R1	15.5 (12.2, 18.7)	−2.5 (−6.4, 1.4)
	≥ 3 R1	12.2 (10.4, 14.1)	−5.3 (−8.2, −2.4)
50th percentile	R0	34.2 (30.8, 37.5)	Reference
	1 R1	29.8 (25.1, 34.5)	−4.6 (−10.2, 1.0)
	2 R1	27.5 (23.3, 31.7)	−6.7 (−11.9, −1.5)
	≥ 3 R1	21.2 (18.1, 24.3)	−12.4 (−16.5, −8.3)
75th percentile	R0	60.7 (52.0, 69.3)	Reference
	1 R1	51.7 (45.0, 58.5)	−9.3 (−19.5, 0.9)
	2 R1	46.0 (38.3, 53.7)	−14.7 (−25.7, −3.7)
	≥ 3 R1	37.5 (32.0, 43.0)	−22.2 (−32.0, −12.4)

Values in parentheses are 95% confidence intervals. PICOS-1 and PICOS-2 are the two study questions based on the PICO framework (see text for details). The following covariates were included in the adjusted model: age at surgery, sex, American Society of Anesthesiologists grade, Eastern Cooperative Oncology Group performance status, body mass index, tumour size, and centre performing the surgery. *Predicted survival times, analysed from time of surgery, represent model-based quantiles (25th, 50th, 75th percentiles). The 95% confidence intervals were obtained using bootstrapped standard errors. †Adjusted difference in predicted survival time between the exposed (number of R1 margins) and unexposed (R0) groups.

## Discussion

This study on 1043 patients from all Swedish centres performing pancreatic surgery found that patients with an R1 status had an adjusted median survival that was 8.2 months shorter than that for patients with an R0 status. Survival was progressively shorter with an increasing number of R1 margins, with the adjusted median survival being was 4.6, 6.7, and 12.4 months shorter for one, two, and three or more R1 margins, respectively, compared with patients in whom all margins were R0.

Previous studies^10,28–32^ have shown an association between R status and overall survival among patients operated on for pancreatic cancer. Attempts have been made to harmonize the definition of R status, and the use of a tumour clearance of 1 mm has become common, although there are indications that a larger distance may be favourable^33^. However, the use of varying pathology protocols hampers comparability between studies^9,12,13^. The issue of different definitions of R status is, in part, a problem with semantics. A recent survey^34^ sent to surgeons and pathologists attempted to understand this. The survey found that there was an agreement between responders that a margin applies only to a border cut by the surgeon (that is, something that the surgeon has technical control of), but there was no consensus as to whether some margins should be defined as margins or anatomical surfaces^34^. This may partially explain some of the differences between protocols. Perceiving R1 mainly as a proxy for surgical technique may bias how R status is defined. The assumption that R1 status primarily reflects surgical performance can be questioned for several reasons. A recent Dutch study^35^ examining the impact of centralizing pancreatic surgery reported a decrease in the rate of R0 resections. Given that centralization aims to concentrate care in high-volume centres, an opposite trend would have been anticipated if margin status were merely a surrogate for surgical expertise. As demonstrated in that study^35^, the observed reduction in R0 rates was at least partly attributable to more meticulous pathological assessment performed by specialized gastrointestinal pathologists. This finding suggests that margin status is influenced not only by surgical technique but also by the rigour and expertise of pathological evaluation. Consequently, classification of R status should be grounded in robust prognostic evidence and derived from standardized pathology assessment and reporting protocols, ensuring consistency and clinical relevance across institutions. The present study found that an R1 status was associated with shorter overall survival. The study originally intended to also investigate whether R1 in specific margins was associated with a disproportionally higher risk of shorter survival. To answer that question in full, a larger cohort would have been needed to isolate the effect of the R status of specific margins. A sensitivity analysis could not identify a higher risk of shorter survival for an R1 status of a specific margin. This finding is similar to that reported previously in a Danish study^33^. The present study found that the number of R1 margins was associated with survival. Previous studies have indicated that the number of margins may play a role in survival, although these studies were on subgroups of patients and were performed in other clinical contexts^36,37^. Although other aspects of the pathological assessment have evolved (for example, it is now recommended that nodal status is reported as pathological (p) N0, pN1, and pN2 rather than a binary outcome^38^), studies analysing the R status beyond a binary classification are lacking. The results from the present study of shorter overall survival for one, two, and three and more R1 margins was found in both the adjusted Laplace regression (overall survival 4.6, 6.7, and 12.4 months, respectively) and the adjusted HR analyses (HR 1.3, 1.4, and 2.0 ,respectively). The progressively shorter survival observed with an increasing number of R1 margins suggests a dose–response relationship between margin involvement and outcome, which is one of the Bradford Hill criteria for inferring potential causality in observational data^39^. This suggests that a more extensive margin involvement may contribute to poorer prognosis. Detecting early recurrence has been shown to be associated with better survival^40^. The results from the present study could suggest that patients with a higher number of R1 margins may benefit from more frequent postoperative follow-up.

There are some limitations to this study that need to be acknowledged. First, there data were missing for recurrence, time to recurrence, and adjuvant chemotherapy. It was not possible to recover these data because the data were obtained from a national registry. However, the median time to recurrence after pancreatic cancer surgery is often reported to be within 12 months^41,42^. Therefore, it is likely that recurrence was also the main cause of death in the present cohort, although local recurrence patterns may be complex and can be driven by several factors. Analysis of recurrence patterns would have addressed this issue; however, the missing data made such analyses impossible, and it was not possible to retrieve these data. Although missing data restrict statistical analysis of recurrence, they do not restrict the analysis of the main outcome, namely overall survival. Regarding missing information on adjuvant chemotherapy, the Swedish national guidelines^16^ recommend that all patients operated on for PDAC are treated with adjuvant chemotherapy, and there is no recommendation that this treatment decision is based on R status. This also applies to recurrent disease. Previous studies^43,44^ have suggested that, in the real world, postoperative complications are one of the greater risk factors for not receiving or tolerating adjuvant chemotherapy. In the present study, there was no significant difference in the rate of postoperative complications between the R0 and R1 groups. This could imply that patients in both groups had similar chances of receiving adjuvant chemotherapy. Even more importantly, adjuvant chemotherapy is not a confounder in the statistical analysis, but a mediator. It should therefore not be adjusted for in the statistical modelling when analysing the full association between R1 and survival. This implies that the lack of these data does not restrict the external validity of the present study.

Second, the assessment of R status may have differed between centres. However, there were no indications during the data quality check that any centre was disproportionally different in their assessment of R status. This implies that the centres followed the national protocol. Third, the definition of R status used in the present study may differ from that used in other studies because a number of definitions exist. Because the Swedish National registry is based on all measured margins, it was not possible to perform subgroup analyses using other protocols without risking systematic confounding. Furthermore, because this study sought to investigate the effect of the number of R1 margins, excluding some margins would have been counterproductive. In the absence of evidence that any specific margin was disproportionately prognostic, these results may demonstrate external validity, implying that the observed association between an increasing number of R1 margins and overall survival could be applicable to other populations of pancreatic adenocarcinoma patients.

In this national register-based study of patients undergoing pancreatoduodenectomy for PDAC, R1 status was associated with an adjusted median overall survival that was 8.2 months shorter than that observed in patients with an R0 status. Moreover, overall survival decreased progressively with an increasing number of R1 margins. The adjusted median survival was 4.6 months shorter for patients with one R1 margin, 6.7 months shorter for those with two R1 margins, and 12.4 months shorter for patients with three or more R1 margins compared with those with an R0 status. These findings indicate that the number of positive margins conveys prognostic information beyond binary R status alone. Accordingly, the current dichotomous reporting of R status may be insufficient, and future guidelines should consider incorporating the number of involved margins into standardized reporting protocols.

## Supplementary Material

zrag155_Supplementary_Data

## Data Availability

Data and analytical methods are available upon reasonable request.

## References

[1] Park W, Chawla A, O'Reilly EM. Pancreatic cancer: a review. JAMA 2021;326:851–862 10.1001/jama.2021.13027 PMC9363152 34547082

[2] Siegel RL, Miller KD, Wagle NS, Jemal A. Cancer statistics, 2023. CA Cancer J Clin 2023;73:17–48 10.3322/caac.21763 36633525

[3] Rahib L, Wehner MR, Matrisian LM, Nead KT. Estimated projection of US cancer incidence and death to 2040. JAMA Netw Open 2021;4:e214708 10.1001/jamanetworkopen.2021.4708 PMC8027914 33825840

[4] Stoop TF, Javed AA, Oba A, Koerkamp BG, Seufferlein T, Wilmink JW et al Pancreatic cancer. Lancet 2025;405:1182–1202 10.1016/S0140-6736(25)00261-2 40187844

[5] Allen PJ, Kuk D, Castillo CF, Basturk O, Wolfgang CL, Cameron JL et al Multi-institutional validation study of the American Joint Commission on cancer (8th edition) changes for T and N staging in patients with pancreatic adenocarcinoma. Ann Surg 2017;265:185–191 10.1097/SLA.0000000000001763 PMC5611666 27163957

[6] Amin MB, Greene FL, Edge SB, Compton CC, Gershenwald JE, Brookland RK et al The Eighth Edition AJCC Cancer Staging Manual: continuing to build a bridge from a population-based to a more ‘personalized’ approach to cancer staging. CA Cancer J Clin 2017;67:93–99 10.3322/caac.21388 28094848

[7] Kalimuthu SN, Wilson GW, Grant RC, Seto M, O'Kane G, Vajpeyi R et al Morphological classification of pancreatic ductal adenocarcinoma that predicts molecular subtypes and correlates with clinical outcome. Gut 2020;69:317–328 10.1136/gutjnl-2019-318217 31201285

[8] Chandrasegaram MD, Goldstein D, Simes J, Gebski V, Kench JG, Gill AJ et al Meta-analysis of radical resection rates and margin assessment in pancreatic cancer. Br J Surg 2015;102:1459–1472 10.1002/bjs.9892 26350029

[9] Leonhardt CS, Niesen W, Kalkum E, Klotz R, Hank T, Buchler MW et al Prognostic relevance of the revised R status definition in pancreatic cancer: meta-analysis. BJS Open 2022;6:zrac010 10.1093/bjsopen/zrac010 PMC8931487 35301513

[10] Delpero JR, Jeune F, Bachellier P, Regenet N, Le Treut YP, Paye F et al Prognostic value of resection margin involvement after pancreaticoduodenectomy for ductal adenocarcinoma: updates from a French prospective multicenter study. Ann Surg 2017;266:787–796 10.1097/SLA.0000000000002432 28953554

[11] Gebauer F, Tachezy M, Vashist YK, Marx AH, Yekebas E, Izbicki JR et al Resection margin clearance in pancreatic cancer after implementation of the Leeds Pathology Protocol (LEEPP): clinically relevant or just academic? World J Surg 2015;39:493–499 10.1007/s00268-014-2808-4 25270344

[12] College of American Pathologists . *Protocol for the Examination of Specimens From Patients With Carcinoma of the Pancreas.* 2021. https://www.cap.org/wp-content/uploads/protocols/Panc.Exo_4.2.0.0.REL_CAPCP.pdf?download=true (accessed 25 August 2026)

[13] The Royal College of Pathologists . *Dataset for Histopathological Reporting of Carcinomas of the Pancreas, Ampulla of Vater and Common Bile Duct*. 2019. https://www.rcpath.org/static/34910231-c106-4629-a2de9e9ae6f87ac1/G091-Dataset-for-histopathological-reporting-of-carcinomas-of-the-pancreas-ampulla-of-Vater-and-common-bile-duct.pdf (accessed 25 August 2026)

[14] von Elm E, Altman DG, Egger M, Pocock SJ, Gøtzsche PC, Vandenbroucke JP et al The Strengthening the Reporting of Observational Studies in Epidemiology (STROBE) statement: guidelines for reporting observational studies. Int J Surg 2014;12:1495–1499

[15] Tingstedt B, Andersson B, Jönsson C, Formichov V, Bratlie SO, Öhman M et al First results from the Swedish National Pancreatic and Periampullary Cancer Registry. HPB (Oxford) 2019;21:34–42 10.1016/j.hpb.2018.06.1811 30097413

[16] Regionala cancercentrum i samverkan . Nationellt vårdprogram bukspottkörtelcancer. 2024. https://kunskapsbanken.cancercentrum.se/diagnoser/bukspottkortelcancer/vardprogram (accessed 25 August 2026)

[17] Tempero MA, Malafa MP, Basturk O, Benson AB, Cardin DB, Chiorean EG et al NCCN Clinical Practice Guidelines in Oncology (NCCN Guidelines^®^) for Pancreatic Adenocarcinoma V.2.2025. © National Comprehensive Cancer Network, Inc. 2025. https://www.nccn.org/guidelines/guidelines-detail?id=1455 (accessed 3 September 2025)

[18] Brown P, Brunnhuber K, Chalkidou K, Chalmers I, Clarke M, Fenton M et al How to formulate research recommendations. BMJ 2006;333:804–806 10.1136/bmj.38987.492014.94 PMC1602035 17038740

[19] Dindo D, Demartines N, Clavien PA. Classification of surgical complications: a new proposal with evaluation in a cohort of 6336 patients and results of a survey. Ann Surg 2004;240:205–213 10.1097/01.sla.0000133083.54934.ae PMC1360123 15273542

[20] Kelly CM, Shahrokni A. Moving beyond Karnofsky and ECOG performance status assessments with new technologies. J Oncol 2016;2016:6186543 10.1155/2016/6186543 PMC4811104 27066075

[21] Hurwitz EE, Simon M, Vinta SR, Zehm CF, Shabot SM, Minhajuddin A et al Adding examples to the ASA-physical status classification improves correct assignment to patients. Anesthesiology 2017;126:614–622 10.1097/ALN.0000000000001541 28212203

[22] Textor J, van der Zander B, Gilthorpe MS, Liskiewicz M, Ellison GT. Robust causal inference using directed acyclic graphs: the R package ‘dagitty’. Int J Epidemiol 2016;45:1887–1894 10.1093/ije/dyw341 28089956

[23] White IR, Royston P, Wood AM. Multiple imputation using chained equations: issues and guidance for practice. Stat Med 2011;30:377–399 10.1002/sim.4067 21225900

[24] Rubin DB . Multiple Imputation for Nonresponse in Surveys. New York: John Wiley & Sons, 1987

[25] Marshall A, Altman DG, Holder RL, Royston P. Combining estimates of interest in prognostic modelling studies after multiple imputation: current practice and guidelines. BMC Med Res Methodol 2009;9:57 10.1186/1471-2288-9-57 PMC2727536 19638200

[26] Pedersen AB, Mikkelsen EM, Cronin-Fenton D, Kristensen NR, Pham TM, Pedersen L et al Missing data and multiple imputation in clinical epidemiological research. Clin Epidemiol 2017;9:157–166 10.2147/CLEP.S129785 PMC5358992 28352203

[27] Bottai M, Zhang J. Laplace regression with censored data. Biom J 2010;52:487–503 10.1002/bimj.200900310 20680972

[28] Demir IE, Jäger C, Schlitter AM, Konukiewitz B, Stecher L, Schorn S et al R0 versus R1 resection matters after pancreaticoduodenectomy, and less after distal or total pancreatectomy for pancreatic cancer. Ann Surg 2018;268:1058–1068 10.1097/SLA.0000000000002345 28692477

[29] Tummers WS, Groen JV, Sibinga Mulder BG, Farina-Sarasqueta A, Morreau J, Putter H et al Impact of resection margin status on recurrence and survival in pancreatic cancer surgery. Br J Surg 2019;106:1055–1065 10.1002/bjs.11115 PMC6617755 30883699

[30] Pine JK, Haugk B, Robinson SM, Darne A, Wilson C, Sen G et al Prospective assessment of resection margin status following pancreaticoduodenectomy for pancreatic ductal adenocarcinoma after standardisation of margin definitions. Pancreatology 2020;20:537–544 10.1016/j.pan.2020.01.004 31996296

[31] Nitta T, Nakamura T, Mitsuhashi T, Asano T, Okamura K, Tsuchikawa T et al The impact of margin status determined by the one-millimeter rule on tumor recurrence and survival following pancreaticoduodenectomy for pancreatic ductal adenocarcinoma. Surg Today 2017;47:490–497 10.1007/s00595-016-1420-7 27677294

[32] Strobel O, Hank T, Hinz U, Bergmann F, Schneider L, Springfeld C et al Pancreatic cancer surgery: the new R-status counts. Ann Surg 2017;265:565–573 10.1097/SLA.0000000000001731 27918310

[33] Aaquist T, Fristrup CW, Hasselby JP, Hamilton-Dutoit S, Eld M, Pfeiffer P et al Prognostic significance of margin clearance in pancreaticoduodenectomy specimens with pancreatic ductal adenocarcinoma in a Danish population-based nationwide study. HPB (Oxford) 2023;25:826–835 10.1016/j.hpb.2023.03.016 37173163

[34] Dhall D, Shi J, Allende DS, Jang KT, Basturk O, Adsay V et al Towards a more standardized approach to pathologic reporting of pancreatoduodenectomy specimens for pancreatic ductal adenocarcinoma: cross-continental and cross-specialty survey from the Pancreatobiliary Pathology Society Grossing Working Group. Am J Surg Pathol 2021;45:1364–1373 10.1097/PAS.0000000000001723 PMC8446290 33899790

[35] Augustinus S, Schafrat PJM, Janssen BV, Bonsing BA, Brosens LAA, Busch OR et al Nationwide impact of centralization, neoadjuvant therapy, minimally invasive surgery, and standardized pathology reporting on R0 resection and overall survival in pancreatoduodenectomy for pancreatic cancer. Ann Surg Oncol 2023;30:5051–5060 10.1245/s10434-023-13465-9 PMC10319672 37210448

[36] Crippa S, Giannone F, Schiavo Lena M, Belfiori G, Partelli S, Tamburrino D et al R status is a relevant prognostic factor for recurrence and survival after pancreatic head resection for ductal adenocarcinoma. Ann Surg Oncol 2021;28:4602–4612 10.1245/s10434-020-09467-6 33393031

[37] Ghaneh P, Kleeff J, Halloran CM, Raraty M, Jackson R, Melling J et al The impact of positive resection margins on survival and recurrence following resection and adjuvant chemotherapy for pancreatic ductal adenocarcinoma. Ann Surg 2019;269:520–529 10.1097/SLA.0000000000002557 29068800

[38] Amin MB, Greene FL, Edge SB, Compton CC, Gershenwald JE, Brookland RK et al AJCC Cancer Staging Manual (8th edn). New York: Springer, 2017 10.3322/caac.21388 28094848

[39] Hill AB . The environment and disease: association or causation? Proc R Soc Med 1965;58:295–300 10.1177/003591576505800503 PMC1898525 14283879

[40] Daamen LA, Groot VP, Besselink MG, Bosscha K, Busch OR, Cirkel GA et al Detection, treatment, and survival of pancreatic cancer recurrence in the Netherlands: a nationwide analysis. Ann Surg 2022;275:769–775 10.1097/SLA.0000000000004093 32773631

[41] Rompen IF, Levine J, Habib JR, Sereni E, Mughal N, Hewitt DB et al Progression of site-specific recurrence of pancreatic cancer and implications for treatment. Ann Surg 2024;280:317–324 10.1097/SLA.0000000000006142 PMC11259998 37870253

[42] Tanaka M, Mihaljevic AL, Probst P, Heckler M, Klaiber U, Heger U et al Meta-analysis of recurrence pattern after resection for pancreatic cancer. Br J Surg 2019;106:1590–1601 10.1002/bjs.11295 31454073

[43] Akahori T, Sho M, Tanaka T, Kinoshita S, Nagai M, Nishiwada S et al Factors associated with failure to complete adjuvant chemotherapy in pancreatic cancer. Am J Surg 2016;211:787–792 10.1016/j.amjsurg.2015.10.034 26846177

[44] Mackay TM, Smits FJ, Roos D, Bonsing BA, Bosscha K, Busch OR et al The risk of not receiving adjuvant chemotherapy after resection of pancreatic ductal adenocarcinoma: a nationwide analysis. HPB (Oxford) 2020;22:233–240 10.1016/j.hpb.2019.06.019 31439478

