# Supplementary material for: Multiple R1 margins are associated with shorter overall survival: national register-based study on pancreatic cancer
Source: BJS Open. 2026 Oct 16;10(5):zrag155. doi: 10.1093/bjsopen/zrag155 (PMC13675251; doi:10.1093/bjsopen/zrag155)
Supplement: Supplementary file 1 [file zrag155_supplementary_data.docx]

**Multiple R1 margins are associated with shorter overall survival: A national register-based study on pancreatic cancer**

Patrik Larsson, MD^1^; Carlos Fernández Moro MD, PhD^2,3^; Melroy D’Souza, MD, PhD^1^; Bobby Tingstedt MD, PhD^4^; Ernesto Sparrelid MD, PhD^1^; Poya Ghorbani MD, PhD^1^

^1^ Division of Surgery and Oncology, Department of Clinical Science, Intervention and Technology, Karolinska Institutet, Karolinska University Hospital, Stockholm, Sweden

^2^ Department of Laboratory Medicine, Division of Pathology F46, Karolinska Institutet, Karolinska University Hospital, Stockholm, Sweden

^3^ Department of Clinical Pathology and Cancer Diagnostics, Karolinska University Hospital, Stockholm, Sweden

^4^ Department of Surgery, Clinical Sciences Lund, Skåne University Hospital, Lund University, Lund, Sweden

**Corresponding author**

Patrik Larsson, MD, PhD candidate

Division of Surgery, Department of Clinical Science Intervention and Technology (CLINTEC), Karolinska Institutet and Center for Digestive Diseases

Karolinska University Hospital K53, 14186 Stockholm, Sweden

E-mail: [patrik.larsson@ki.se](mailto:patrik.larsson@ki.se)

**Supplementary Materials - Index**

| **Supplementary Tables and Figures** |  |
| --- | --- |
| Table S1 | *page 3* |
| Table S2 | *page 4* |
| Table S3 | *page 5* |
| Table S4 | *page 6* |
| Table S5 | *page 7* |
| Table S6 | *page 8* |
| Figure S1 | *page 9* |
| Figure S2 | *page 10* |
| **References** | *page 11* |

| **Table S1**. Histopathological characteristics | | | |
| --- | --- | --- | --- |
|  | Overall (n=1043) | R0 (n=398) | R1 (n=645) |
| No. of regional lymph nodes resected | 21 [16–27] | 21 [15–27] | 21 [16–27] |
| Missing, n (%) | 67 (6.4) | 29 (7.3) | 33 (5.1) |
| No. Of regional metastatic lymph nodes | 3 [1–6] | 2 [1-5] | 4 [2–7] |
| Missing, n (%) | 67 (6.4) | 29 (7.3) | 33 (5.1) |
| No. of nonregional lymph nodes resected | 0 [0–3] | 0 [0–3] | 1 [0–3] |
| Missing, n (%) | 88 (8.4) | 48 (12.1) | 43 (6.7) |
| No. of nonregional metastatic lymph nodes | 0 [0–0] | 0 [0–0] | 0 [0–1] |
| Missing, n (%) | 89 (8.5) | 48 (12.1) | 44 (6.8) |
| Tumour size total (mm) | 34 [27–42] | 30 [25–40] | 35 [28–43] |
| Missing, n (%) | 81 (7.8) | 39 (9.8) | 41 (6.4) |
| Values for continuous data are median [IQR] and count (%) for number of patients. | | | |

| **Table S2**. Number of R1, n (%) | |
| --- | --- |
|  | Overall (n=1043) |
| R0 | 398 (38.2) |
| 1 R1 | 252 (24.2) |
| 2 R1 | 161 (15.4) |
| 3 R1 | 97 (9.3) |
| 4 R1 | 49 (4.7) |
| 5 R1 | 25 (2.4) |
| 6 R1 | 16 (1.5) |
| 7 R1 | 5 (0.5) |
| 8 R1 | 1 (0.1) |
| R1, Missing number of margins | 39 (3.7) |

| **Table S3**. Background characteristics for patients stratified by the number of R1 or R0 | | | | | | |  |
| --- | --- | --- | --- | --- | --- | --- | --- |
|  | Overall (n=1043) | R0 (n=398) | 1 R1(n=252) | 2 R1(n=161) | ≥3 R1 (n=193) | R missing§ (n=39) |  |
| Age, years* | 71 [65–77] | 71 [64–76] | 71 [64–77] | 71 [64–76] | 73 [67–77] | 71 [60–77] |  |
| Sex, women* | 532 (51.0) | 196 (49.2) | 135 (53.6) | 77 (47.8) | 101 (52.3) | 23 (59.0) |  |
| BMI, kg/m^2^ | 25 [22 - 28] | 25 [23–28] | 22 [20–28] | 25 [22–28] | 25 [22–27] | 25 [23–27] |  |
| Missing, n (%) | 26 (2.5) | 13 (3.3) | 6 (2.4) | 3 (1.9) | 1 (0.5) | 3 (7.7) |  |
| ASA-score* | 2 [2–3] | 2 [2–3] | 2 [2–3] | 2 [2–3] | 2 [2–3] | 2 [2–2] |  |
| Missing | 5 (0.5) | 0 | 3 (1.2) | 0 | 1 (0.5) | 1 (2.6) |  |
| ECOG score* | 0 [0–1] | 0 [0–1] | 1 [0–1] | 0 [0–1] | 0 [0–1] | 0 [0–1] |  |
| Missing, n (%) | 12 (1.2) | 0 | 5 (2.0) | 1 (0.6) | 5 (2.6) | 1 (2.6) |  |
| CA19-9 (kU/l) | 168 [48–557] | 125 [34–405] | 181 [45–519] | 175 [67–922] | 208 [57–697] | 151 [47–517] |  |
| Missing, n (%) | 216 (20.7) | 105 (26.4) | 49 (19.4) | 26 (16.1) | 24 (12.4) | 12 (30.8) |  |
| Peroperative bleeding* | 350 [200–600] | 350 [200–600] | 325 [200–600] | 300 [200–600] | 350 [200–650] | 500 [300–1000] |  |
| Missing, n (%) | 4 (0.4) | 0 | 1 (0.4) | 0 | 0 | 3 (7.7) |  |
| Venous resection | 243 (23.3) | 35 (8.8) | 68 (27.0) | 38 (24) | 100 (52) | 2 (5.1) |  |
| Missing, n (%) | 75 (7.2) | 41 (10.3) | 1 (0.4) | 1 (0.6) | 0 | 32 (82.1) |  |
| Artery resection | 4 (0.4) | 1 (0.3) | 2 (0.8) | 0 | 1 (0.5) | 0 |  |
| Missing, n (%) | 158 (15.1) | 78 (19.6) | 21 (8.3) | 20 (12.4) | 20 (10.4) | 19 (48.7) |  |
| Tumour size total (mm) | 34 [27–42] | 30 [25–40] | 32 [25–38] | 35 [29–41] | 40 [31–50] | 32 [24–35] |  |
| Missing, n (%) | 81 (7.8) | 39 (9.8) | 5 (2.0) | 3 (1.9) | 1 (0.5) | 33 (84.6) |  |
| Values for continuous data are median [IQR] and count (%) for number of patients. §There were 39 patients classified as R1 but with no R status information on the specified margins. Abbreviations: ASA, American society of anaesthesiologists’ physical status; BMI, body mass index; ECOG, eastern cooperative oncology group performance status; CA 19-9 serum carbohydrate antigen 19-9. | | | | | | |  |

| **Table S4**. Outcomes stratified by R0 and the number of R1 | | | | | | | |
| --- | --- | --- | --- | --- | --- | --- | --- |
|  | Overall (n=1043) | R0 (n=398) | 1 R1(n=252) | 2 R1(n=161) | ≥3 R1 (n=193) | R missing§ (n=39) | p-value |
| Dead | 708 (67.9) | 245 (61.6) | 165 (65.5) | 113 (70.2) | 148 (76.7) | 37 (94.9) | 0.002 |
| Recurrence | 328 (31.4) | 112 (28.1) | 72 (28.6) | 54 (33.5) | 73 (37.8) | 17 (43.6) | 0.021 |
| Missing, n (%) | 245 (23.5) | 89 (22.4) | 63 (25.0) | 42 (26.1) | 48 (24.9) | 3 (7.7) | - |
| CD ≥IIIA | 197 (18.9) | 71 (17.8) | 54 (21.4) | 33 (20.5) | 31 (16.1) | 8 (20.5) | 0.457 |
| Missing, n (%) | 11 (1.1) | 2 (0.5) | 3 (1.2) | 2 (1.2) | 4 (2.1) | 0 | - |
| §There were 39 patients classified as R1 but with no R status information on the specified margins. p-values were calculated with χ^2^ test, comparing margins to R0. Abbreviations: CD, Clavien-Dindo grade. | | | | | | | |

| **Table S5.** Cox proportional hazard regression analysis for overall survival from time of surgery | | | | | |
| --- | --- | --- | --- | --- | --- |
|  |  | HR (95% CI) unadjusted | p-value | HR (95% CI) adjusted* | p-value |
|  | **R0** | Ref | Ref | Ref | Ref |
|  | **R1** | 1.47 (1.36–1.72) | <0.001 | 1.55 (1273– 1.79) | <0.001 |
|  | **R0** | Ref | Ref | Ref | Ref |
|  | **1 R1** | 1.24 (1.02–1.52) | 0.030 | 1.29 (1.05–1.60) | 0.019 |
|  | **2 R1** | 1.38 (1.10–1.73) | 0.005 | 1.44 (1.15–1.82) | 0.002 |
|  | **≥3 R1** | 1.98 (1.61–2.43) | <0.001 | 1.94 (1.55–2.42) | <0.001 |
| *The following covariates were included in the adjusted model: age at surgery, sex, ASA, ECOG, BMI, tumour size, centre performing the surgery. Abbreviations: ASA, American Society of Anaesthesiologists’ classification system; ECOG, Eastern Cooperative Oncology Group performance status; BMI, Body Mass Index; 95% CI, 95 % confidence interval; HR, Hazard Ratio. | | | | | |

| **Table S6.** Cox proportional hazard regression analysis for overall survival from time of surgery analysed with multiple imputation | | | | | |
| --- | --- | --- | --- | --- | --- |
|  |  | HR (95% CI) unadjusted | p-value | HR (95% CI) adjusted* | p-value |
|  | **R0** | Ref | Ref | Ref | Ref |
|  | **R1** | 1.47 (1.34–1.72) | <0.001 | 1.61 (1.42– 1.94) | <0.001 |
|  | **R0** | Ref | Ref | Ref | Ref |
|  | **1 R1** | 1.23 (1.02–1.52) | 0.028 | 1.43 (1.14–1.59) | 0.003 |
|  | **2 R1** | 1.44 (1.13–1.72) | 0.004 | 1.52 (1.23–1.92) | <0.001 |
|  | **≥3 R1** | 2.01 (1.64–2.41) | <0.001 | 2.04 (1.70–2.49) | <0.001 |
| Multiple imputation was performed using chained imputation. The imputation model included the exposure (resection margin), all confounders (sex, age at surgery, centre performing the surgery, BMI, ASA, ECOG performance status, tumour size), auxiliary variables (positive lymph nodes, previous ERC, preoperative bilirubin, preoperative CA 19-9, peroperative bleeding), the outcome (survival status) and log-transformed follow-up time. The imputation was generated with 20 imputed datasets and combined effect estimates using Rubin’s rules.  *The following covariates were included in the adjusted model: age at surgery, sex, ASA, ECOG, BMI, tumour size, centre performing the surgery. Abbreviations: ASA, American Society of Anaesthesiologists’ performance status; ECOG, Eastern Cooperative Oncology Group performance status; BMI, Body Mass Index; 95% CI, 95 % confidence interval; HR, Hazard Ratio. | | | | | |

**Figure S1.** Kaplan-Meier analysis of overall survival stratified by R0 and the number of R1 from time of surgery

Subheading: All p-values are derived from the log rank test, with R0 as reference

**Figure S2.** Directed acyclic graphs (DAG)

Subheading: Directed acyclic graph (DAG) illustrating the authors’ understanding of the potential causal relationship between R status (green) and overall survival (blue with I). DAG is a method for visualising potential causal pathways, including exposure, outcome, confounders and mediators. The DAG was made using DAGitty software developed by Johannes Textor et al. ^1^

**References**

1.	Textor J, van der Zander B, Gilthorpe MS, Liskiewicz M, Ellison GT. Robust causal inference using directed acyclic graphs: the R package 'dagitty'. *Int J Epidemiol*. Dec 1 2016;45(6):1887-1894. doi:10.1093/ije/dyw341
